# Effect of *Bacillus subtilis* and *Paenibacillus polymyxa* on the Compressive Strength and Self-Healing of Type IP Concrete

**DOI:** 10.3390/ma19112277

**Published:** 2026-05-28

**Authors:** Baruc Ronel Rivas Torres, Olenka Guibell Mendoza Tejada, Rubén Francisco Gamarra Tuco, Yuma Ita-Balta, Fernando Farfán-Delgado, Cecilia Manrique-Sam

**Affiliations:** 1 School of Civil Engineering, Universidad Católica de Santa María, Arequipa 04013, Peru; baruc.rivas@ucsm.edu.pe (B.R.R.T.); ruben.gamarra@ucsm.edu.pe (R.F.G.T.); 2 School of Industrial Engineering, Universidad Católica de Santa María, Arequipa 04013, Peru; olenkamt28@gmail.com; 3 Faculty of Pharmaceutical, Biochemical and Biotechnological Sciences, Universidad Católica de Santa María, Arequipa 04013, Peru; yita@ucsm.edu.pe; 4 School of Human Medicine, Universidad Católica de Santa María, Arequipa 04013, Peru; ffarfand@ucsm.edu.pe

**Keywords:** bacteria, bioconcrete, calcium carbonate, self-healing concrete, compressive strength, *Bacillus subtilis*, *Paenibacillus polymyxa*, biorepair, microcracks

## Abstract

The influence of *Bacillus subtilis* (Solution A) and *Paenibacillus polymyxa* (Solution B) bacteria on the properties of conventional concrete with a design compressive strength of f′c = 210 kg/cm^2^ and on the repair of microcracks and fissures was evaluated. Yura Type IP and Frontera Type IP cements were used, together with aggregates from the Chiguata and La Poderosa quarries (Arequipa, Peru). Two mix design methods were applied: ACI 211 and the fineness modulus of the combined aggregates. For microcrack repair, injections of Solutions A and B were applied, followed by either water curing or curing in the corresponding bacterial solution. For water replacement, both solutions were used at concentrations of 10%, 15%, and 20%. Compressive strengths were measured at 7, 14, 21, and 28 days. The results indicate that bacterial incorporation, together with reductions in the effective water-to-cement ratio associated with bacterial solution replacement, was associated with improvements in compressive strength and microcrack repair through mechanisms consistent with calcium carbonate (CaCO_3_) precipitation. For the injection group, a maximum strength of 196.09 kg/cm^2^ was obtained. For the water replacement group, a maximum strength of 335.71 kg/cm^2^ was reached, representing a 59.9% increase over the standard design. The *P. polymyxa* solution consistently outperformed *B. subtilis* across all groups and concentrations evaluated. These findings suggest that bacterial solutions—particularly *P. polymyxa*—may represent a promising complementary strategy to improve concrete performance and durability under the evaluated experimental conditions.

## 1. Introduction

The use of concrete has grown exponentially in Latin America and worldwide; therefore, the development of sustainable concrete is essential, particularly for environmental reasons. Currently, 7–8% of anthropogenic CO_2_ emissions in the atmosphere are attributed to cement production and processing, which drives the search for mechanisms to prolong the service life of concrete structures, making the material more durable and sustainable [1].

Concrete is one of the most widely used construction materials in the world, relatively economical and broadly employed. Its main limitation is the formation of cracks, which can arise from multiple causes: low tensile strength, shrinkage, deficient materials, inadequate construction practices, and exposure conditions. Large cracks significantly reduce structural integrity and durability. Small cracks in the early stages do not immediately alter strength, but over time, they affect porosity and permeability, facilitating the ingress of corrosive agents that deteriorate the concrete and cause corrosion of the steel reinforcement [2].

An optimal and viable solution is the self-healing of concrete. The bacterial precipitation of calcium carbonate (CaCO_3_) is highly useful for this purpose: it is more compatible with the concrete mix and favorable for the environment [3]. Various studies have evaluated methods for incorporating bacteria as self-healing agents, including encapsulation, direct addition, and replacement of mixing water [4]. Bacteria of the genus *Bacillus* have shown the greatest response in the self-healing process. *P. polymyxa* has been considerably less studied in self-healing concrete applications compared with *Bacillus* spp., despite its known biomineralization and environmental adaptation capabilities [5].

Recent studies have also highlighted the importance of microstructural optimization strategies in improving the mechanical performance and durability of cementitious materials. For example, multiscale characterization approaches applied to nano-modified geopolymers have demonstrated that enhanced particle dispersion and microstructural refinement can significantly influence mechanical behavior and crack resistance. These findings support the relevance of investigating complementary physicochemical mechanisms that may contribute to performance improvements in bio-based concrete systems.

In the Peruvian context, previous research has explored the use of bacteria of the genus *Bacillus* in concrete. Mendoza & Sánchez (2017) reported favorable results with *B. subtilis* in Cusco [6]; Asenjo (2019) analyzed the influence of bacterial additive on controlled cracking in Cajamarca [7]; and Santos (2021) applied *B. subtilis* in the settling tanks of the La Atarjea water treatment plant in Lima [8]. However, comparative studies involving multiple mix design methods, cement types, and local quarries from Arequipa are scarce. The present study aims to evaluate the effect of *B. subtilis* and *P. polymyxa* on the compressive strength of f′c = 210 kg/cm^2^ concrete with induced fissures, using two bacterial incorporation mechanisms, two design methods, two Type IP cements, and two aggregate sources, with a complementary cost analysis.

It was hypothesized that the incorporation of bacterial solutions, particularly *P. polymyxa*, would enhance compressive strength and promote microcrack self-healing through mechanisms associated with microbially induced calcium carbonate precipitation, with the magnitude of the effect depending on the incorporation method and replacement percentage.

## 2. Theoretical Background

### 2.1. Biorepair and Microbially Induced Calcium Carbonate Precipitation (MICP)

The biorepair agent is a mixture of solutions containing bacterial spores that serve as intermediaries in synthesizing calcium carbonate from calcium lactate and calcium nitrate, thereby converting it into calcite (CaCO_3_), which can repair fissures up to 1 mm wide [9]. Bacterial calcium carbonate precipitation (MICP) is an effective means of sealing fissures with calcite crystals, extending concrete durability and reducing repair and maintenance costs, thereby contributing to a reduction in CO_2_ emissions [10]. Calcium nitrate-based bacteria produce self-repaired concrete with strength superior to that of typical concrete [11]. The simplified reaction is:

Ca(C_3_H_5_O_3_)_2_ + 6O_2_ → CaCO_3_ + 5CO_2_ + 5H_2_O

Specimens that exhibited higher axial compressive strength accumulated more calcium carbonate crystals, suggesting a direct relationship between biomineralization and mechanical strength improvement [12].

### 2.2. Bacterial Strains Used

***Bacillus subtilis*** (Solution A—strain ATCC 11774): Gram-positive bacillus, 0.5–2.5 × 1.2–10 μm, motile, with highly resistant oval endospores. Reproduction temperature: ~35 °C in 23 h. Culture concentration used: 1.6 × 10^8^ spores/L with calcium nitrate 80 g/L [9]. Its application in concrete fissures has demonstrated efficacy in sealing cracks up to 0.3 mm [13].

***Paenibacillus polymyxa*** (Solution B—strain ATCC 842): Gram-variable, facultatively aerobic, endospore-forming bacillus. Optimal pH 7.0 (maximum 10.5–11), optimal temperature 30–40 °C. Produces metabolites with applications in bioremediation, bioflocculation, and mineral processing. Culture concentration: 1.6 × 10^8^ spores/L with calcium nitrate 80 g/L [14].

### 2.3. Mix Design Methods

Two methods were used: the ACI 211.1 Method, based on the water-to-cement ratio principle, which follows a series of ordered steps to obtain the weights and volumes of each material per 1 m^3^ of concrete [15]; and the Fineness Modulus of the Aggregate Combination Method, which performs mix design primarily considering the fineness modulus of fine and coarse aggregates. The fineness modulus is an indicator of specific surface area: as aggregate fineness increases, paste demand increases and bond strength decreases [16].

## 3. Materials and Methods

The objective of the research is to determine the influence of incorporating *Bacillus subtilis* and *Paenibacillus polymyxa* bacteria on the carbonation, compressive strength, and self-healing of microcracks and fissures in concrete, using different proportions of bioremediation solution via two methods: injection and replacement of design water.

### 3.1. Aggregate Properties and Bacterial Culture

Aggregates constitute between 60% and 75% of concrete volume; therefore, their type and quality decisively influence both fresh and hardened mix properties. Tests were conducted on aggregates from the Chiguata Quarry and La Poderosa Quarry to determine properties such as granulometric analysis, moisture content, specific gravity, absorption, unit weight, and abrasion. Table 1 summarizes material characterization.

For the microbiological component, bacterial strains were acquired from KWIK-STIK Laboratories through BHIOS Laboratorios S.R.L., Arequipa, Peru. The microorganisms used were *Bacillus subtilis* strain ATCC 11774 and *Paenibacillus polymyxa* strain ATCC 842. The viability of each strain was evaluated by reconstituting the cultures under sterile conditions and inoculating them onto nutrient agar plates, followed by incubation at 35 °C for 24 h. Colony-forming units (CFU) were quantified by plate counting to verify the target bacterial concentration.

Bacterial stress tolerance was preliminarily evaluated through exposure to adverse environmental conditions, including abrupt temperature changes up to 120 °C, pH variations, and nutrient deprivation conditions. These tests were performed to assess the tolerance and potential survival capacity of the bacterial strains under conditions associated with cementitious environments.

### 3.2. Biorepair Solution

The biorepair agent was obtained by mixing the bacterial culture concentrate (1.6 × 10^8^ spores/L, verified by plate count) with calcium nitrate (80 g/L). Two solutions were produced: Solution A (*B. subtilis* + calcium nitrate) and Solution B (*P. polymyxa* + calcium nitrate). These solutions can repair fissures up to 1 mm wide by synthesizing calcite (CaCO_3_) [9]. Bacterial carbonate precipitation also contributes to reducing concrete porosity and improving long-term durability [10].

### 3.3. Concrete Production and Experimental Design

The target population comprised 1248 cylindrical specimens (diameter 10 cm, height 20 cm) with f′c = 210 kg/cm^2^, divided into two groups: the first cracked with the application of a biorepair solution by injection, and the second with different percentages of design water replaced by the solution. Table 2 presents the complete distribution of experimental designs.

### 3.4. Statistical Analysis

For each experimental group and testing age, compressive strength was determined as the mean of three specimens. Results are reported as mean ± standard deviation (SD). A factorial analysis of variance (ANOVA) was performed to evaluate the effects of the main factors (bacterial solution type, incorporation method, replacement percentage, cement type, and aggregate source) and their interactions on 28-day compressive strength. Assumptions of normality (Shapiro–Wilk test) and homogeneity of variances (Levene’s test) were verified prior to ANOVA. Post hoc pairwise comparisons were conducted using Tukey’s HSD test. Differences were considered statistically significant at *p* < 0.05. All statistical analyses were performed using Minitab 19 (Minitab LLC, State College, PA, USA).

### 3.5. Injection Process

Specimens were subjected to controlled cracking on the fifth day after casting by applying a compressive load equivalent to approximately 60% of the estimated 5-day compressive strength until visible surface fissures appeared. This age was selected to promote the formation of early-stage fissures while maintaining sufficient structural integrity for subsequent bacterial treatment and mechanical evaluation. The selected load level was intended to induce controlled surface microfissures without causing complete specimen failure.

To improve reproducibility, all specimens were subjected to the same loading protocol under identical testing conditions. Crack widths were immediately measured using a crack comparator gauge, and only specimens presenting crack widths within the predefined experimental range (0.1–0.8 mm) were included in the self-healing evaluation. The induced cracks were predominantly surface microfissures generated along the central region of the cylindrical specimens. Specimens were subsequently injected with the corresponding biorepair solutions at ages 0, 7, 14, and 21 days. The injected solution volume was adjusted according to fissure extent and penetration capacity. After injection, specimens continued under normal curing conditions and were tested for compressive strength at 7, 14, 21, and 28 days of age.

### 3.6. Water Replacement Process

Specimens were prepared by replacing a percentage (10%, 15%, or 20%) of the design water with the biorepair solution, following standard curing procedures, and tested at 7, 14, 21, and 28 days of age.

### 3.7. Compressive Strength Testing and Microscopic Observation

Compression tests were conducted at the Civil Engineering Laboratory of Universidad Católica de Santa María in accordance with NTP 339.034 and ASTM C-39. The average strength per group (3 specimens per evaluation point) was calculated. Calcium carbonate crystal observation was performed on cross-sections of the best-performing specimens using a stereoscope (ED.1402-P EduBlue, Euromex Microscopen BV, Arnhem, The Netherlands) at variable magnifications (×7, ×10, ×20, ×30, and ×40). Bacterial self-healing of fissures in cementitious matrices depends on crack size and the curing protocol applied [17].

## 4. Results

### 4.1. Compressive Strength of Standard Concrete

**Figure 1:**
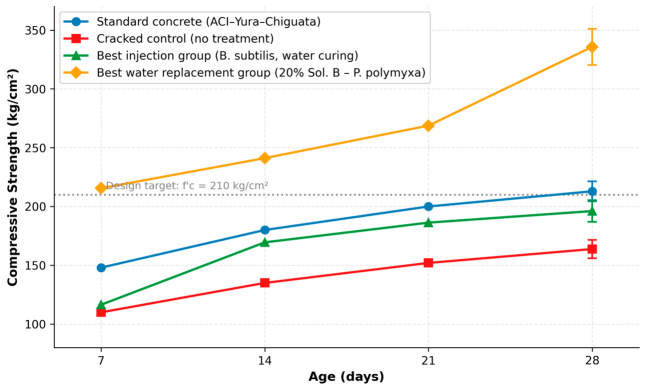
Compressive strength development over time for the main experimental groups. Dashed line: design target f′c = 210 kg/cm^2^.

Standard concrete satisfactorily met the design f′c in all evaluated groups. The maximum standard strength was 238.18 kg/cm^2^ (Design 5: ACI—Yura—La Poderosa), 13.4% above the design target. Table 3 presents the average 28-day compressive strengths with standard deviations. Figure 1 shows the compressive strength development over time for the main experimental groups.

### 4.2. Effect of Cracking on Compressive Strength

Controlled crack induction on the fifth day of curing significantly reduced strength across all groups (*p* < 0.001, ANOVA). For the ACI–Yura–Chiguata group, strength dropped from 212.9 ± 8.5 to 163.8 ± 7.8 kg/cm^2^ at 28 days (−23.1%). Average damaged area ranged from 7.5% to 15% of the cross-sectional area. As a result of this investigation, fissures up to 0.3 mm were satisfactorily repaired; fissures up to 0.5 mm were partially repaired; and fissures larger than 0.5 mm could not be repaired with the applied protocols.

### 4.3. Results of the Injection Group

**Figure 2:**
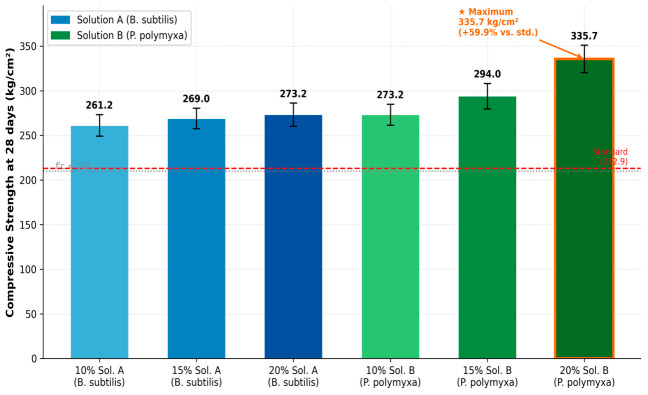
Maximum compressive strength at 28 days for water replacement groups. ★ Maximum strength of the entire investigation.

The best results in the injection group were obtained with Solutions A and B cured in water. Specimens injected and cured in bacterial solution showed decreases below expected values at all ages in almost all cases; therefore, this protocol is not recommended for microcrack repair. Optimum compressive strengths for the injection group at 7, 14, 21, and 28 days were 116.45, 169.50, 186.26, and 196.09 kg/cm^2^, respectively. Recent studies confirm that the efficiency of the injection method depends on the subsequent curing protocol and the bacterial concentration applied [18,19]. Table 4 presents the comparative results for the ACI–Yura–Chiguata group. Figure 2 compares the compressive strength at 28 days for the injection group specimens.

### 4.4. Results of the Water Replacement Group

**Figure 3:**
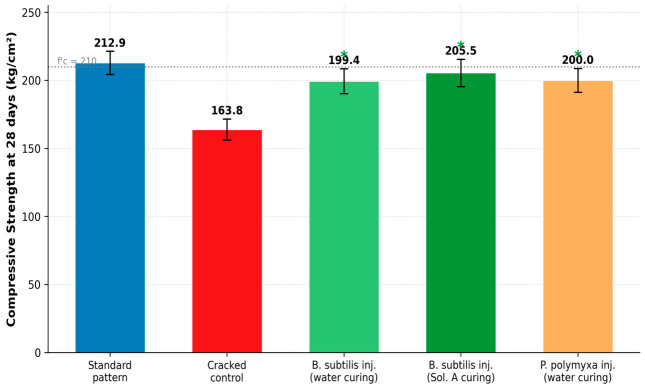
Compressive strength at 28 days for injection group comparison (ACI–Yura–Chiguata). * *p* < 0.05 vs. cracked pattern (Tukey’s HSD).

The highest compressive strengths were obtained in the water-replacement groups. As the replacement percentage increased, so did the strength increment. Optimum compressive strengths at 7, 14, 21, and 28 days for the water replacement group were 215.55, 241.11, 268.74, and 335.71 kg/cm^2^, respectively. This behavior is consistent with studies on ecological bioconcrete that report improvements in strength when bacteria are incorporated directly into the mix [20]. Bacterial water replacement acts similarly to certain admixtures, altering the effective *w*/*c* ratio and improving concrete microstructure [21]. Table 5 presents the best designs per group. Figure 3 presents the maximum compressive strengths obtained for the water replacement groups.

To achieve Slump values of 2–4″ (design values), the required water was less than the design water as the replacement percentage increased. This resulted in a decrease in the *w*/*c* ratio, which in turn led to an increase in strength. The greater the percentage of bacterial solution replacement, the greater the increase in strength.

Although part of the strength increase can be explained by the reduction in the water-to-cement ratio according to Abrams’ law, the remaining improvement may be associated with bacterial activity, including mechanisms consistent with CaCO_3_ precipitation and pore refinement. Comparable effects associated with bacterial calcium carbonate precipitation and durability enhancement have been previously described [22]. However, this contribution could not be independently isolated in the present study.

Separation of the *w*/*c* effect from the bacterial effect. Because the bacterial solution replacement reduced the effective *w*/*c* ratio, it is important to distinguish the strength gain attributable to this reduction from the gain due to bacterial activity per se. Using Abrams’ law and the control group’s strength–*w*/*c* relationship as a baseline, the estimated strength increases due solely to the *w*/*c* reduction for Design 94 (*w*/*c* = 0.5158 vs. the standard *w*/*c* = 0.5617) is approximately +15.4%. However, the total observed increase was +59.9%, indicating that a substantial portion of the strength gain may be associated with bacterial activity and microstructural modifications; however, this contribution cannot be quantitatively isolated with the present experimental design.

### 4.5. Observation of Crystal Formations Consistent with Calcium Carbonate

Cross-sections of the specimens with the best strength increments were observed using a stereoscope at variable magnifications (×7 to ×40). Crystal formations consistent with calcium carbonate were observed filling pores and microfissures. Water replacement specimens exhibited more abundant crystal formations than injected specimens. Specimens with higher compressive strength presented greater crystal accumulation, suggesting a possible association between biomineralization and mechanical strength [12]. It should be noted that crystal identification was performed by optical stereoscopy only; confirmation of the mineralogical composition (e.g., by SEM-EDS or XRD analysis) was beyond the scope of this study and is recommended for future investigations.

### 4.6. Cost Analysis

Experimental designs with bacterial solutions yield costs comparable to or lower than those of equivalent conventional designs. The bacterial solution cost represents only S/. 0.07 to S/. 0.14 per m^3^ (at 10% to 20% concentrations). Design 94 achieved the best cost-benefit ratio (0.99) with a compressive strength of 335.71 kg/cm^2^. Maintaining the cement quantity while increasing the bacterial solution concentration reduces the water required per design, making bioconcrete economically competitive against conventional repair solutions [23].

## 5. Discussion

The experimental results suggest that the incorporation of bacterial solutions may contribute to improvements in compressive strength compared with standard designs. Similar improvements in bacterial concrete containing *Bacillus subtilis* have also been reported in previous experimental studies [24]. The highest strengths were consistently observed in the water replacement groups rather than in the injection groups. Among the evaluated strains, Solution B (*P. polymyxa*) outperformed Solution A (*B. subtilis*) in all experimental conditions. Previous studies using *Bacillus subtilis* have similarly reported improvements in crack sealing and durability properties in cementitious matrices [25].

The factorial ANOVA confirmed that both the type of bacterial solution (F = 18.7, *p* < 0.001) and the replacement percentage (F = 42.3, *p* < 0.001) had statistically significant main effects on 28-day compressive strength, as did their interaction (F = 5.2, *p* = 0.008). This result is consistent with the literature, indicating that bioremediation efficacy depends on local environmental conditions, pH of the medium, and cement composition [11]. The superiority of *P. polymyxa* may be attributed to the alkaline pH of the curing environment in Arequipa, which favors its optimal growth (pH 7.0–10.5) [14,26].

The water replacement mechanism proved more effective than injection. The effectiveness of bacterial incorporation into the concrete matrix has also been observed in studies using bacterial consortiums for crack control applications [27]. The homogeneous distribution of bacterial spores throughout the concrete mass may generate additional nucleation sites for CaCO_3_, not only in fracture zones [12,28]. This is consistent with the concept of bioconcrete, in which bacteria are an integral part of the mix from its preparation [5,29]. Specimens with higher strength formed greater crystal accumulation, suggesting a possible relationship between biomineralization and mechanical strength.

Regarding the *w*/*c* ratio, as the bacterial solution replacement percentage increased, the water required to achieve the target slump (2–4″) was lower than the original design water content. This resulted in a reduction in the effective water-to-cement ratio, which likely contributed to the observed increase in compressive strength. As discussed in Section 4.4, the Abrams’ law analysis was used only as a theoretical comparative reference to estimate the possible contribution of the *w*/*c* reduction. Based on this approximation, approximately 15.4% of the total 59.9% strength increase observed in Design 94 could be associated with the reduced *w*/*c* ratio. The remaining increase may be related to additional mechanisms, including bacterial activity and microstructural modifications; however, these contributions could not be independently isolated or quantitatively validated under the present experimental design.

During mix preparation, it was also observed that increasing the replacement percentage modified physical properties such as ductility and setting time. Additional studies incorporating higher replacement percentages and controlled *w*/*c* conditions would be necessary to better characterize these effects [21]. The influence of supplementary cementitious materials and mix optimization on self-healing performance has also been emphasized in recent reviews [30].

Study limitations include the absence of permeability and long-term durability tests, the use of optical stereoscopy rather than SEM/XRD for crystal identification, the relatively small sample size per testing point (n = 3), and the absence of a non-bacterial control group with an equivalent reduction in the effective water-to-cement ratio. Consequently, the independent contribution of bacterial activity and physicochemical effects associated with reduced mixing water could not be quantitatively isolated under the present experimental design. Self-healing efficiency in cementitious matrices varies with time and exposure conditions [17,18].

Future research should evaluate bacterial viability after prolonged storage, optimize nutrient type (calcium lactate vs. calcium citrate), confirm CaCO_3_ crystal composition by SEM-EDS or XRD, assess additional mechanical properties (modulus of elasticity, tensile strength), incorporate controlled reduced *w*/*c* reference groups, and conduct pilot-scale testing in real structural elements.

## 6. Conclusions

1. The incorporation of *Bacillus subtilis* and *Paenibacillus polymyxa* was associated with improvements in compressive strength and microcrack sealing, potentially through mechanisms consistent with calcium carbonate precipitation.
2. The water replacement method showed higher performance than the injection method, reaching a maximum compressive strength of 335.71 kg/cm^2^ at 28 days under the evaluated experimental conditions.
3. Among the evaluated bacterial strains, under the evaluated experimental conditions, *P. polymyxa* showed higher compressive strength values compared with *B. subtilis*.
4. Although part of the observed strength increase may be explained by the reduction in the effective water-to-cement ratio, the results suggest that additional mechanisms potentially associated with bacterial activity and microstructural modifications may also have contributed. However, these effects could not be independently isolated or quantitatively validated under the present experimental design.
5. Crystal formations morphologically consistent with CaCO_3_ were observed; however, their mineralogical composition was not confirmed, representing a limitation of the study.
6. The increase in bacterial solution replacement percentage improved the cost–benefit ratio, suggesting potential economic feasibility under the evaluated conditions.

Therefore, the observed improvements should be interpreted as the result of combined physicochemical and biological effects rather than a purely biological mechanism.

## Figures and Tables

**Table 1:** Material characterization—Aggregate and cement properties.

Property	La Poderosa	Unit	Chiguata	Unit	Chiguata B2	Unit	Material
Sp. gravity Yura IP	2.83	g/cm^3^	2.83	g/cm^3^	2.83	g/cm^3^	Cement
Sp. gravity Frontera IP	2.89	g/cm^3^	2.89	g/cm^3^	2.89	g/cm^3^	Cement
Specific gravity (fine)	2.58	g/cm^3^	2.43	g/cm^3^	2.43	g/cm^3^	Fine agg.
Absorption (fine)	1.49	%	1.27	%	0.97	%	Fine agg.
Fineness modulus (fine)	2.62	—	2.74	—	2.99	—	Fine agg.
Loose unit wt. (fine)	1505	kg/m^3^	1364.9	kg/m^3^	1201.1	kg/m^3^	Fine agg.
Specific gravity (coarse)	2.64	g/cm^3^	2.30	g/cm^3^	2.50	g/cm^3^	Coarse agg.
Absorption (coarse)	0.62	%	1.77	%	0.72	%	Coarse agg.
Fineness modulus (coarse)	6.31	—	6.94	—	6.79	—	Coarse agg.
Nominal max. size	1/2″	—	1/2″	—	1/2″	—	Coarse agg.

Note: Specific gravity values for cement and aggregates are reported as apparent specific gravity (dimensionless ratio expressed in g/cm^3^).

**Table 2:** Distribution of experimental groups and designs.

Experimental Group	Chiguata ACI	Chiguata FM	La Poderosa ACI/FM	No. of Designs
Control pattern	1–2	3–4	5–8	8
Cracked pattern (control)	9–10	11–12	13–16	8
Exp. Subtilis injection—water curing	17–18	19–20	21–24	8
Exp. Polymyxa injection—water curing	25–26	27–28	29–32	8
Exp. Subtilis injection—Sol. A curing	33–34	35–36	37–40	8
Exp. Polymyxa injection—Sol. B curing	41–42	43–44	45–48	8
Exp. water replacement 10%—Sol. A	49–50	51–52	53–56	8
Exp. water replacement 15%—Sol. A	65–66	67–68	69–72	8
Exp. water replacement 20%—Sol. A	81–82	83–84	85–88	8
Exp. water replacement 10%—Sol. B	57–58	59–60	61–64	8
Exp. water replacement 15%—Sol. B	73–74	75–76	77–80	8
Exp. water replacement 20%—Sol. B	89–90	91–92	93–96	8
TOTAL				96 designs/1248 specimens

FM = Fineness Modulus method. 12 specimens per design (3 per testing age). Note: The numbering sequence corresponds to the original laboratory coding system assigned during specimen preparation and does not indicate omitted experimental groups.

**Table 3:** Average compressive strength of standard concrete at 28 days (kg/cm^2^).

Method	Cement	Quarry	f′c 28 d ± SD (kg/cm^2^)	CV (%)	Meets f′c = 210
ACI 211	Yura IP	Chiguata	212.9 ± 8.5	4.0	Yes
ACI 211	Frontera IP	Chiguata	233.9 ± 10.2	4.4	Yes
ACI 211	Yura IP	La Poderosa	238.2 ± 9.1	3.8	Yes
ACI 211	Frontera IP	La Poderosa	233.9 ± 11.0	4.7	Yes
Fineness Mod.	Yura IP	Chiguata	163.8 ± 7.8	4.8	Ref. *
Fineness Mod.	Frontera IP	Chiguata	147.7 ± 6.5	4.4	Ref. *

SD = standard deviation (n = 3). CV = coefficient of variation. * Included as a comparative reference to evaluate the bacterial effect.

**Table 4:** Compressive strength comparison at 28 days: standard, cracked, and experimental injection (ACI–Yura–Chiguata).

Group	f′c 28 d ± SD (kg/cm^2^)	Δ vs. Cracked	Δ vs. Standard
Standard pattern	212.9 ± 8.5	—	—
Cracked pattern (control)	163.8 ± 7.8	—	−23.1%
Exp. *B. subtilis* injection (water curing)	199.4 ± 9.2	+21.8% *	−6.3%
Exp. *B. subtilis* injection (Sol. A curing)	205.5 ± 10.1	+25.5% *	−3.5%
Exp. *P. polymyxa* injection (water curing)	200.0 ± 8.8	+22.1% *	−6.0%

* Statistically significant vs. cracked pattern (*p* < 0.05, Tukey’s HSD).

**Table 5:** Best compressive strengths per replacement group and experimental *w*/*c* ratio.

Group	Max. f′c ± SD (kg/cm^2^)	Optimal Design	Exp. *w*/*c*	H_2_O adj. (mL)	Δ vs. Std.	Δ Abrams Est.
Repl. 10% Sol. A	261.2 ± 12.1	D51: FM–Yura–Chig.	0.5543	−40	+23.7% *	+8.2%
Repl. 15% Sol. A	269.0 ± 11.5	D69: ACI–Yura–LP	0.5411	−180	+27.9% *	+10.5%
Repl. 20% Sol. A	273.2 ± 13.0	D86: FM–Front.–LP	0.5261	−340	+29.8% *	+12.8%
Repl. 10% Sol. B	273.2 ± 11.8	D64: FM–Front.–LP	0.5355	−240	+29.7% *	+11.2%
Repl. 15% Sol. B	294.0 ± 14.2	D77: ACI–Yura–LP	0.5242	−360	+39.9% *	+13.1%
Repl. 20% Sol. B ★	335.7 ± 15.3	D94: ACI–Front.–LP	0.5158	−450	+59.9% *	+15.4%

★ Maximum strength of the entire investigation. FM = Fineness Modulus method. LP = La Poderosa. * Statistically significant vs. standard (*p* < 0.05). Δ Abrams est. = estimated strength increase attributable solely to the reduction in *w*/*c* ratio.

## Data Availability

The original contributions presented in this study are included in the article. Further inquiries can be directed to the corresponding author.
